# Isolation, characterization and antifungal docking studies of wortmannin isolated from *Penicillium radicum*

**DOI:** 10.1038/srep11948

**Published:** 2015-07-10

**Authors:** Vineeta Singh, Vandana Praveen, Divya Tripathi, Shafiul Haque, Pallavi Somvanshi, S. B. Katti, C. K. M. Tripathi

**Affiliations:** 1 Microbiology Division, CSIR-Central Drug Research Institute, Sitapur Road, Lucknow-226031, Uttar Pradesh, India; 2 Fermentation Technology Division, CSIR-Central Drug Research Institute, Sitapur Road, Lucknow-226031, Uttar Pradesh, India; 3 Division of Organic Chemistry, CSIR - National Chemical Laboratory, Pune- 411008, Maharashtra, India; 4 Department of Biosciences, Jamia Millia Islamia (A Central University), New Delhi-110025, India; 5 Centre for Drug Research, Faculty of Pharmacy, Viikki Biocentre-2, FI-00014, University of Helsinki, Helsinki, Finland; 6 Department of Biotechnology, TERI University, New Delhi-110070, India; 7 Medicinal and Process Chemistry Division, CSIR-Central Drug Research Institute, Sitapur Road, Lucknow-226031, Uttar Pradesh, India

## Abstract

During the search for a potent antifungal drug, a cell-permeable metabolite was isolated from a soil isolate taxonomically identified as *Penicillium radicum.* The strain was found to be a potent antifungal agent. Production conditions of the active compound were optimized and the active compound was isolated, purified, characterized and identified as a phosphoinositide 3-kinase (PI3K) inhibitor, commonly known as wortmannin (Wtmn). This is very first time we are reporting the production of Wtmn from *P. radicum.* In addition to its previously discovered anticancer properties, the broad spectrum antifungal property of Wtmn was re-confirmed using various fungal strains. Virtual screening was performed through molecular docking studies against potential antifungal targets, and it was found that Wtmn was predicted to impede the actions of these targets more efficiently than known antifungal compounds such as voriconazole and nikkomycin i.e. 1) mevalonate-5-diphosphate decarboxylase (1FI4), responsible for sterol/isoprenoid biosynthesis; 2) exocyst complex component SEC3 (3A58) where Rho- and phosphoinositide-dependent localization is present and 3) Kre2p/Mnt1p a Golgi alpha1,2-mannosyltransferase (1S4N) involved in the biosynthesis of yeast cell wall glycoproteins). We conclude that Wtmn produced from *P. radicum* is a promising lead compound which could be potentially used as an efficient antifungal drug in the near future after appropriate structural modifications to reduce toxicity and improve stability.

The need for safe and effective antifungal drugs has increased in parallel with the expansion of immunocompromised patients at risk for fungal infections, along with the emergence of multi-resistant fungal strains all over the world. The targets of the currently available antifungal compounds are also found in mammalian cells, which leads to toxicity and/or adverse drug interaction in the host. The major limitations associated with currently used antifungal compounds are low efficacy rate and severe side effects. Thus, it is imperative to expedite the search for a potent antifungal compound which must be non-toxic to mammalian cells and can be employed as an alternative for classical antifungal drugs^1^.

Previous findings reported that several fungal strains are capable of producing narrow to broad spectrum antifungal metabolites e.g., *Penicillium radicum*, *Rhizoctonia solani, Fusarium oxysporum, Phytophthora cinnamomi, Pleiochaeta setosa, Plasmodiophora brassicae, Verticillium dahliae, Pythium ultimum, Sclerotium rolfsii* etc. An earlier report showed that *P. radicum* can be used as an effective fungicide^2^. *Penicillium* spp. have ubiquitous occurrence in diverse ecological niches and they demonstrate some important functional attributes associated with plant growth, such as, solubilisation of phosphorus, biological control of root diseases and phytohormone production^3^. Several metabolites from *Penicillium* spp., such as paxisterol and compactin, have been reported for analgesic and antifungal properties in animals^4^.

The most common fungal pathogens for humans continue to be the species of *Candida* and *Aspergillus*. For many years, the treatment of invasive fungal infections was limited to amphotericin B. Afterwards, two triazoles (voriconazole and posaconazole) and three echinocandins (anidulafungin, caspofungin and micafungin) have been mostly used for the treatment of fungal infections^5^. Emergence of resistance and a high mortality rate in candidiasis and aspergillosis warrants for discovery of new antifungal drug targets or strategies or agents in antifungal therapy.

Wortmannin (Wtmn), a cell-permeable fungal metabolite, has been identified as a potent, selective and irreversible inhibitor (IC_50_ = 4.2 nM) of phosphatidylinositol 3-kinase (PI3K)^6^. There is evidence of the involvement of PI3K in several intracellular signaling pathways, like Toll-like receptor (TLR) signaling^7^. Inhibition of PI3K with Wtmn enhances TLR-mediated inducible nitric-oxide synthase (iNOS) expression, activates NF-κB and up-regulates cytokine mRNA production^8^. Also, PI3K is required for autophagy^9^. Autophagy is a complex pathway in which cell material can be sequestered and delivered to the lysosome for degradation. Inhibition of PI3K with Wtmn can inhibit autophagic sequestration^9^. Wtmn is a potent anti-cancer drug which failed clinical trials due to problems related to toxicity, solubility, and stability^10^. Subsequently, it was re-formulated as a nano-particle to solve all the above mentioned issues that earlier caused its failure^11^. Earlier reports also suggested that slight structural modifications in Wtmn distant from the furan ring had little or no effect on its *in-vitro* efficacy^12^, this finding opened the door for the commercial use of Wtmn and its analogues in medical applications and warrants for conformational/interaction understanding of the compound.

Molecular docking plays an important role in structure-based drug designing, functional sites prediction on protein molecular surfaces, protein ligand docking etc^13^,^14^. Molecular docking attempts to predict the binding mode by evaluating the energy scores of different bound conformations with a scoring function. Ligand-binding sites are among the most promising targets for drug candidates, whose actions depend upon the inhibition or regulation of the target protein functions. Ligand based methods use the shape similarity concept while the structure based methods rely on scoring functions i.e., reverse/inverse docking against a panel of targets^15^.

To the best of our knowledge, isolation of Wtmn from *Penicillium radicum* has not been reported earlier (STN search by SciFinder). In the present study, we describe the isolation, identification, characterization, and antifungal and anticancer activities of Wtmn from *P. radicum* isolated from a soil sample collected from agricultural field of northern India. Also, *in-silico* molecular docking studies have been done to predict the possible antifungal targets for the active metabolite.

## Results and Discussion

### Producer organism and fermentation profile

**Figure 1:**
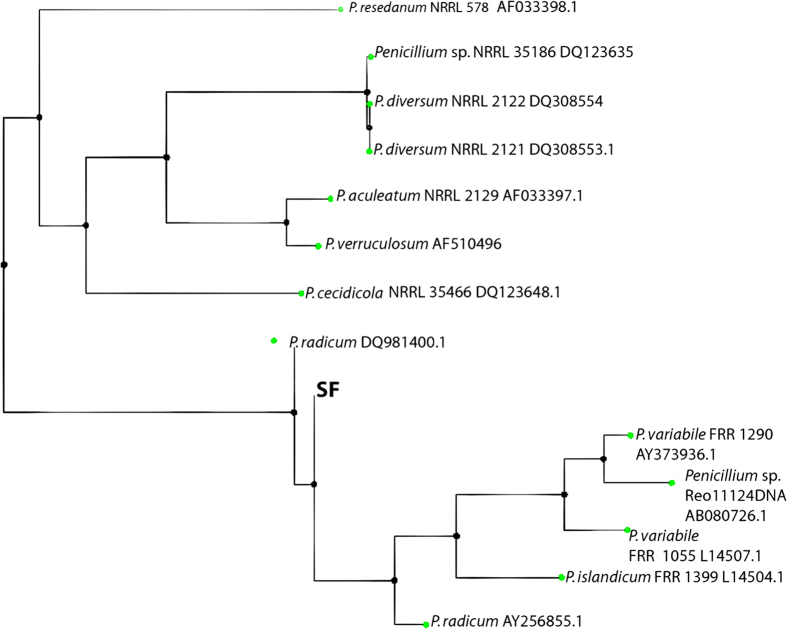
Phylogenetic relationship between *Penicillium radicum* [SF] and other *Penicillium* sp. 2.

On the basis of morphological, cultural and physiological characteristics, the isolated strain was found to be closely related to ascomycetes (Table 1 and Fig. 1). This fungal strain was designated as SF and further identified on the basis of 18S rRNA homology studies and the sequence was submitted in the GenBank under accession no. KJ528407. A partial 18S rRNA gene sequence (1112 bp in length) of SF was compared with the sequences already available in the GenBank database through BLAST analysis and found to possess close similarity to some members of the class ascomycetes, like, *Penicillium radicum* (DQ891400.1), *P. radicum* (AY256855.1), *P. variablie* (AY3739393.1), *P. islandicum* (L14504.1) and *Penicillium* sp. Re 011124 (AB 080726.1) (Fig. 1). Finally, the neighbour-joining phylogenetic tree of the partial 18S rRNA sequence data confirmed that *P. radicum* (DQ891400.1) was most closely related to the strain SF with 100% sequence similarity (Fig. 1). Olive-green in coloured ellipsoidal conidia with roughened or spiral-striated walls (5 × 3.75 μm) were observed under scanning electron microscope (Supplementary Information: Figure SI1). The strain was submitted in Microbial Type Culture Collection (MTCC), Institute of Microbial Technology (IMTECH) (www.http://mtcc.imtech.res.in), Chandigarh, Punjab, India as *Penicillium radicum* MTCC 7584.

**Figure 2:**
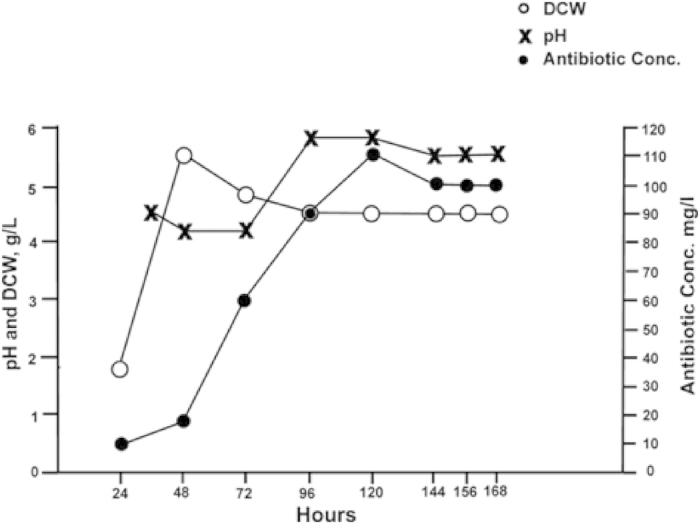
Fermentation profile of *Penicillium radicum.*

The growth and fermentation profile of SF (*P. radicum*) was evaluated in Potato dextrose broth (PDB) medium and results have been summarized in Fig. 2. The log phase was observed between 20–48 h and maximum biomass (5.5 g/L) was found at 48 h of the fungal growth. The production of the antifungal metabolite commenced in the late log phase of the fungal growth and accumulated maximum (110 mg/L) in the stationary phase (approximately 120 h of the incubation) and decreased slowly, afterwards. The pH of the production medium decreased slightly during the growth of the fungus, whereas in the production phase it was increased from 4.0 to 5.5, which suggests that the extracellular metabolites formed during the late log phase might be of basic nature, hence increased the pH of the medium.

### Purification and chemical characterization of the active compound

The antibiotic (crude) productivity purified through gradient elution with silica gel column (liquid-liquid extraction) was 500 mg/L. The fractions showing antifungal activity were pooled together (185 mg) and again subjected to silica gel column chromatography. Finally, the active fractions were subjected to high performance liquid chromatography (HPLC) to confirm the purity of the active compound (Supplementary Information: Figure SI2). The active compound (approximately 88% pure) was eluted at 11.74 min as mentioned in the material and methods section (Supplementary Information; Figure SI2). Overall, 110 mg of SF-1 (active compound) was recovered from the supernatant of 1L fermented culture. The active compound constituted about 22% of the ‘crude’ obtained from *P. radicum.*

The crystals of the purified antifungal compound were ‘off-white’ in color and decomposed at 240–241 °C. The powder of the purified compound was hygroscopic and very sensitive towards light. When stored under sealed and moisture free condition at −20 °C, it remained active for 4–6 months. The solubility of the active compound was in the order of these solvents, i.e., CHCl_3_ > ethyl acetate > MeOH > acetonitrile. The molecular formula determined through elemental analysis was found to be C_23_H_24_O_8_ (data not shown). It was further confirmed by electrospray ionization mass spectrometry (ESI-MS), ^1^H and ^13^C nuclear magnetic resonance (NMR). ESI-MS analysis showed an intense ion peaks at m/z 451 (M + Na^+^) and 879 (2M+ Na^+^) and revealed that the molecular weight of the compound was 428 (Supplementary Information; Figure SI3). The UV/visual absorption spectrum showed an intense band at λ = 205 nm due to π→π^*^ or n→π^*^ transition (Supplementary Information; Figure SI4). The band pattern of the isolated compound (λ = 205, 256 and 292 nm) showed similarity to polyene compounds and suggested a polyene nature of the antifungal compound^16^ (Supplementary Information; Figure SI4). The presence of the ether (1215 and 1,230), ester (1,745 and 1,750) and carbonyl (1,675 and 1,680) groups in the chemical structure of the compound was confirmed by its Infrared (IR) spectrum (KBr)^17^ (Supplementary Information; Figure SI3).

^13^C NMR (δ) ppm; ^1^H NMR (δ) ppm (multiplicity, J) (Supplementary Information; Figure SI5 & Table S1) : C_1_CH 88.518; 4.76 (dd, 1.83, 6.96), C_2_ CH_2_ 72.870 ; H_A_: 3.46 (dd, 1.95. 11.23) H_B_: 3.01 (dd,7.01, 11.09), C_3_ CO 127.569, C_4_ C 114.276, C_5_ C 142.853, C_6_ C 144.777, C_7_ O 172.628, C_8_ C 140.371, C_9_ C 149.550, C_10_ C 40.758 , C_11_ CH 70.060; 6.16 (ddd, 2.77, 7.53, 8.93), C_12_ CH_2_ 36.153; H^A^: 2.59 (m) H^B^: 1.60 (dd,8.7, 12.8), C_13_ C 49.181, C_14_ CH 44.080; 2.89 (ddd, 2.66, 5.97, 12.68), C_15_ CH_2_ 22.926; H^A^: 3.17 (m) H^B^: 2.06 (m), C_16_ CH_2_ 35.710; H^A^: 2.59 (m) H^B^: 2.26 (m); C_17_ CO 216.150, C_18_ CH_3_ 14.564; 0.97 (s), C_19_ CH_3_ 26.492; 1.74 (s), C_20_ CH 149.970; 8.25 (s), C_21_ CO 169.482, C_22_ CH 21.017; 2.14 (s), C_23_ CH 59.437; 3.19 (s).

A search with the spectroscopic data (^1^H, ^13^C-NMR, HMBS, HSQC and H2BC) in AntiBase [A natural products database for rapid structure determination. Chemical Concepts, Weinheim, Germany (2000)] showed similarity with PI3K inhibitor, Wtmn. The structure was finally confirmed by comparing ^13^C and ^1^H-NMR data (Supplementary Information; Figure SI5 & Table SI1) with the literature^18^ and it was found to be identical with Wtmn. Wtmn is a hydrophobic estrogen-related metabolite and the production of Wtmn has been reported earlier in *Talaomyces/ Penicillium wortmannii, Penicillium funiculosum, Myrothecium roridum* and *Fusarium oxysporum*^19^,^20^,^21^,^22^,^23^. The chemical structure of Wtmn was similar to the structure deposited at PubChem compound database under CID [312145], which was further used for *in silico* antifungal target prediction studies.

### Bioactivity of the active compound

The purified active compound obtained from SF culture was established as Wtmn. Earlier, Brian *et al.* (1957) observed the antifungal activity of Wtmn against thirty-eight fungal species (*Acrosialagmus cinnabarinus, Alternaria* sp*., Aspergillus claoatus, A. Flavus, A. terreus, A. niger, A. Sydowi, Botrytis allii, B. cinerea, B. fabae, Cladosporium herbarum, Rhizopus stolonifer, Cephalosporium acremonium, Fusarium bulbigenum, F. graminearum, Stachybotrys atra, Stemphylium* sp.) and found strong activity against five species (*Botrytis allii, B. cinerea, B. fabae, Cladosporium herbarum and Rhizopus stolonifer*)^19^. However, in the present study, we extended the bioactivity profile of Wtmn by testing the antifungal property against various strains of *Candida*, *Cryptococcus terreus, Trichophyton rubrum, Rhizoctonia oryzae, Aspergillus versicolor, Aspergillus fumigates, Aureobasidium pullulans, Fusarium moniliforme,* and *Saccharomyces cerevisiae*.

Previous findings reported that the members of Eurotiales of class Eurotiomycetes and hypocreales of Sordariomycetes of ascomycota division of fungal kingdom are capable of producing Wtmn. Among four reported Wtmn producers^19^,^20^,^21^,^22^,^23^, two *Talaromyces wortmannii* and *Penicillium funiculosum* belong to Trichocomaceae family. *Talaromyces funiculosus* is the anamorph of *Penicillium funiculosum*. *Talaromyces* species and most species of *Penicillium* subgenus Biverticillium reside in a monophyletic clad distant from species of other subgenera of *Penicillium*^24^. *Penicillium radicum* is also reported to belong to the subgenus *Biverticillium* and has affinities with *P. variabile, P. allahabadense* and *Talaromyces wortmannii*, but can be distinguished by differences in morphology, secondary metabolite profiles and unique DNA banding patterns^25^. However, it may be possible that horizontal gene transfer from *Talaromyces wortmannii* which is the first known producer of Wtmn to *P. funiculosum* and *P. radicum* is responsible for Wtmn production.

The broad spectrum antifungal activity of Wtmn was re-confirmed against 18 different unicellular as well as filamentous fungal strains and it was found that Wtmn demonstrated excellent antifungal activities. The detailed findings of antifungal screening have been given in Table 2. Wtmn’s MIC value spanned a relatively wide range for both unicellular and filamentous fungi ranging from 0.39 μg/ml (for *C. albicans* ATCC 24433) to 25 μg/ml (for *A. niger* DSM 2182). Wtmn showed either better or equivalent and in minor cases modest antifungal activities in comparison to other commercially available antifungal drugs (amphotericin B and fluconazole) against 18 fungal strains as mentioned in the Table 2.

Earlier *in-vitro* studies reported that Wtmn has good anticancer activity^26^, though its clinical use for anticancer chemotherapy is restricted due to its toxicity and low stability of the furan ring in solutions^12^. On the similar lines, the cytotoxic activity of the isolated Wtmn was evaluated against four human tumor cell lines under *in-vitro* conditions (Table 3). The cytotoxicity assay showed that the most sensitive lines were HCT-15 and 502713 colon carcinoma and they showed 75 and 90% inhibition at Wtmn concentrations of 10 and 30 μg/ml, respectively. Generally, Wtmn showed modest activity in low passage human breast cancer cell lines. Wtmn was capable of inhibiting about 52% of HEP-2 liver carcinoma at higher concentrations (30 μg/ml). Interestingly, IMR-32 neuroblastoma cells were fully insensitive to Wtmn (Table 3).

The *in-vivo* anti-inflammatory and immunosuppressive effects of Wtmn suggested that it has a potential to work as an inhibitor of signal transduction pathways. Wtmn interacts with many biological targets, but binds *in-vitro* most strongly to PI3K^12^. Wtmn’s electrophilic 21-site makes a covalent linkage with the enzyme and the inhibition of PI3K is irreversible^12^. The PI3K enzyme is part of a signalling cascade that is essential for cell growth and differentiation, therefore Wtmn is a potent antiproliferative agent. Despite the toxicity issues, the nanomolar level inhibition of PI3K by Wtmn has inspired numerous synthetic and biological studies for its modification as low toxic lead compound^12^.

### Antifungal target prediction for Wtmn

Wtmn is known to interact with many biological targets, such as PI3K, mammalian Polo like kinase (PLK1), and serine/threonine kinases of the PI3K family, and has been shown to have biological activity, including antifungal and anti-inflammatory properties^12^. Selectivity and excessive toxicity is the major obstacle in the *in-vivo* clinical use of Wtmn. However, previous findings suggested that slight structural changes in Wtmn has no major effect on its efficacy and prompted us to conduct antifungal target site prediction studies via molecular docking for structural and conformational understanding which can be exploited for lead development.

Interactions of Wtmn with the targets of cancerous cells have been studied very well but its interactions with antifungal targets have not been studied yet. Thus, to elucidate these unexplored ‘interactions’, virtual screening was performed through *in silico* molecular docking studies for the prediction of the target sites for Wtmn. For this, Wtmn was compared with the previously known potential antifungal compounds that can bind to the active site of proteins, thus, obstructing the activities of pathogenicity. The structural changes in proteins are responsible for various biological functions and keeping this fact in view, the antifungal potential of Wtmn was examined through its interaction with the target proteins. Investigations on the atomic and residual properties of the isolated Wtmn and other protein molecules used in the present study were retrieved with the help of dedicated software programs. The *in silico* analysis showed that the structure of Wtmn perfectly bound with the active sites of known predicted target proteins, viz. 1FI4, 1S4N and 3A58, and was then compared with the binding of other known antifungal compounds, like, voriconazole, nikkomycin, and pyridobenzimidazole (Table 4). 1FI4 is responsible for sterol/isoprenoid biosynthesis, 3A58 where Rho- and phosphoinositide-dependent localization is present and 1S4N is involved in the biosynthesis of yeast cell walls glycoproteins^27^,^28^,^29^. Our antifungal docking studies demonstrated that Wtmn bound more effectively with above mentioned targets than the known antifungal compounds, i.e., voriconazole and nikkomycin.

The comparative studies of interactions of known antifungal compounds under consideration and Wtmn with the above predicted targets were performed on the basis of several energies, namely docking energy, intermolecular energy, torsional energy, root mean square (RMS) and internal energy and it was found that the docking energies of Wtmn were greater than the known antifungal compounds (Table 5). In protein-compound interactions, the lower the docking energy, the higher the binding affinity. The binding free energy evaluation by AutoDock (open source software program AutoDock 4.2) includes intermolecular energy (van der Waals energy, hydrogen bonding energy, desolvation energy, and electrostatic energy), internal energy and torsional energy. The first two items build up docking energy; the first and third items compose binding energy. During *in silico* analysis the difference in the binding free energy of three different classes of inhibitors provides further insights into their binding mode with protein structure. Our *in silico* docking results demonstrated that Wtmn was energetically favoured over voriconazole for all three predicted target proteins, while pyridobenzimidazole was highly favoured for the target protein IS4N and slightly better for the other two targets i.e., 1FI4 & 3A58 (Table 5).

**Figure 3:**
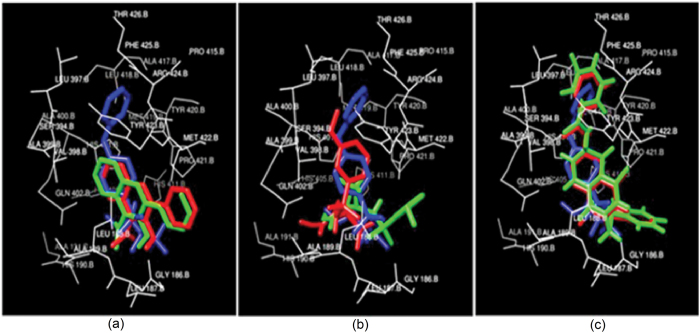
Docked conformations of Inhibitors in Red (AutoDock) and Green (FlexiDock) with respect to crystal structure ligand in blue. (**a**) Wortmannin (**b**) Voriconazole (**c**) Pyridobenzimidazole.

**Figure 4:**
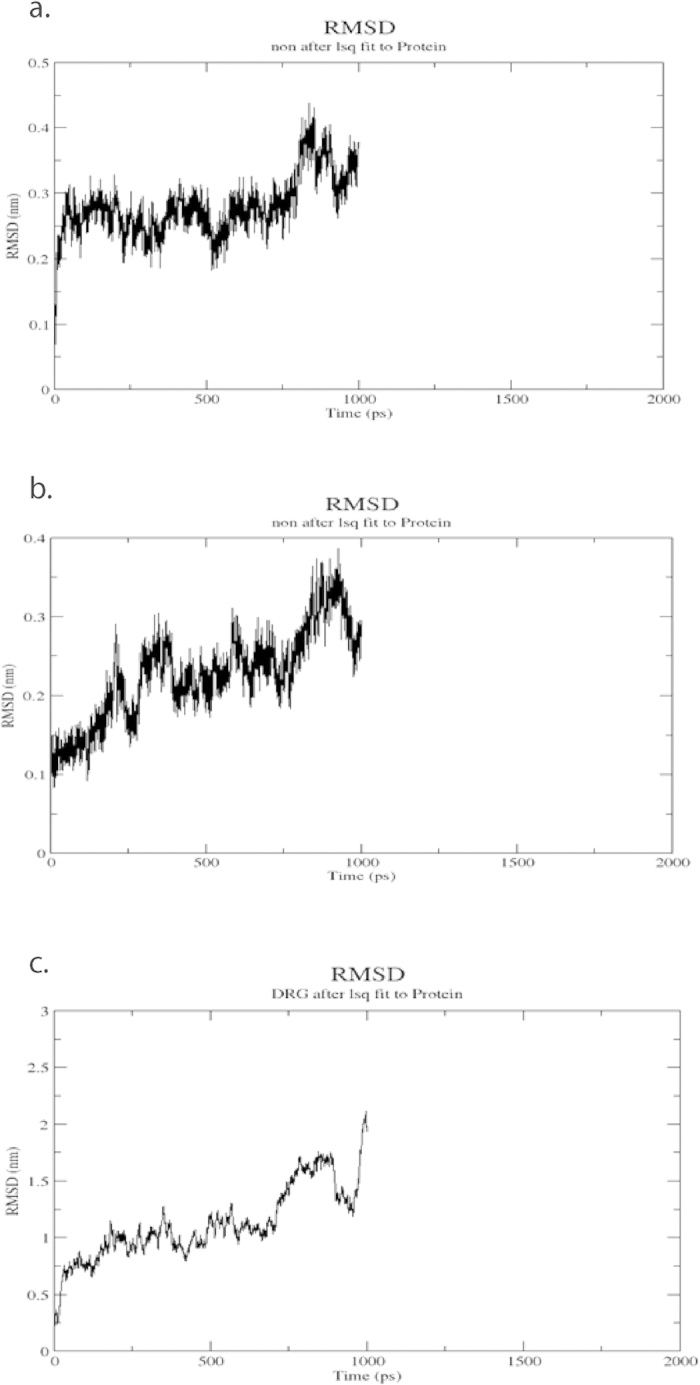
Root mean squared deviations (RMSD) between - **(a)** Wortmannin and backbone atoms of Protein structure (PDB ID: 1S4N) **(b)** Pyridobenzimidazole and backbone atoms of Protein structure (PDB ID: 1FI4) **(c)** Voriconazole and backbone atoms of Protein structure (PDB ID: 3A58).

Docking studies showed that the amino acid residues of enzyme 1FI4, viz. THR262, SER258, MSE255, VAL229 and LEU221 were observed to play an active role in the interaction with Wtmn. However, it is a known target site for antifungal compound voriconazole where amino acid residues ASP205, ALA303, GLY304, ASP201, GLY112, SER207 were found to be active. Likewise, the *in silico* analysis also showed that 1S4N amino acid residues, such as, SER382, TYR380, HIS379, ILE378, LEU431, GLN429 were active with Wtmn, whereas ASP383, HIS379, GLU396, TYR426, LEU431, SER382 were active against pyridobenzimidazole, which is a well-known compound of this predicted target site. Similarly, for 3A58 the active amino acid residues for Wtmn were GLU103, ASP98, CYS93, LYS100, ASN106 whereas ARG96, PRO105, GLU103, ASP98, ILE166, CYS92, ASP98 were active against nikkomycin (Table 6). Due to the presence of these interactions with these active amino acid residues, Wtmn worked more efficiently than the existing antifungal compounds studied in the present computational study. The computational docking technique has already been successfully used in predicting targets for the development of drug against KAS II of *Mycobacterium tuberculosis* H37Rv^30^ and virulent proteins (aerolysin and hemolysin) of *Aeromonas hydrophila*^31^,^32^. Our docking study also revealed that amino acid residues, SER258 and ALA303 of 1FI4 play an active role during interaction with Wtmn and known antifungal drug voriconazole (Fig. 3). Similarly, the amino acid residues SER382, TYR380 for Wtmn and SER382 for Pyridobenzimidazole were active during interaction with the 1S4N (Fig. 3); and those involved in interaction with 3A58 were ASN106, CYS93 for Wtmn and ARG96, CYS93 against nikkomycin (Fig. 4). No hydrogen bond was formed between those compounds as high interaction energy was observed. Earlier, the active amino acids’ residues were reported to be targeted with varied drugs to inhibit DNA ligase of *M. tuberculosis*^33^, oligomerization in *A. hydrophila*^34^, through robust binding affinity between protein and drug interactions. The twenty five docking conformations for each ligand were divided into separate conformational clusters according to 2 Ǻ RMSD criteria. AutoDock ranked each conformational cluster by binding free energy evaluation to find the best binding mode^35^. Best ranking conformational clusters from each class of ligand were further docked using FlexiDock (Licenced version software program) module in SYBYL-X (SYBYL X. Tripos International 1699 South Hanley Rd; St.Louis, Missouri, 63144, USA) and have been shown in Table 7. Results from AutoDock and FlexiDock showed agreement with less than 2 Ǻ RMSD deviations in the conformation of docked ligands (Fig. 3). We have examined the mobility of the inhibitors in the active site through 1ns (1000 ps) MD simulation. This was done by calculating the RMSD of inhibitors atom position to antifungal backbone for all the three systems under consideration. A comparison of RMSD deviation and subsequent conformational changes indicated binding mobility in all the three groups of inhibitor.

For pyridobenzimidazole, in the first 450 ps of simulation, the inhibitor aligned itself to a more horizontal position and subsequently loosens contact with catalytic dyad subsite resulting in opening up of active site cleft (Fig. 4b). However, about halfway through the simulation inhibitor anchored again itself to a catalytic dyad. This occurred by anchoring with the side chains of Asp383 and His379 and finally closing the active site. This conformation gradually stabilized at about 800 ps with slight changes in the backbone structure. For voriconazole, the inhibitor changed its original binding conformation and aligned itself in the catalytic dyad. The active site pocket achieved an open conformation while the inhibitor further buried itself in the catalytic dyad. This resulted in enhanced interaction of statine moiety with Ala 303 and brought about slight closing of the active site cleft (Fig. 4c), that conformation failed to attain stability and remained unstable for the rest of the simulation. However, for Wtmn, the binding conformation of the inhibitor forced the active site to remain in a closed conformation for most of the period. Still, around 250 ps the inhibitor realigned itself in the catalytic dyad further closed the active site to attain very tight binding conformation, and this conformation remained stable for rest of the simulation (Fig. 4a).

In the present *in silico* study, molecular docking and molecular dynamics approaches were applied to study the binding conformations and structural specificity of the antifungal inhibitors. Voriconazole and pyridobenzimidazole compounds demonstrated similar binding orientations with general specificity towards the catalytic dyad of the enzyme. Pyridobenzimidazole anchored more steadily into the catalytic dyad by having more hydrogen bonding pairs between the enzyme and the inhibitor. Moreover, in computational studies, Wtmn showed better binding and greater specificity than the other two antifungal compounds, which have the ability to anchor both catalytic dyad residues and flap residues thereby maintaining a tightly closed conformation throughout the simulation period, which indicated greater specificity towards the structure of the enzyme. Our *in silico* study explores structural specificity of all the three inhibitors towards the available protein structures and provides valuable insight into designing of highly specific inhibitors towards antifungal enzyme. Overall, Wtmn demonstrated promising antifungal results and future studies related to experimental validation of predicted target(s) through enzyme assay could provide excellent leads for highly selective inhibitors for antifungal enzyme. Currently, our group and collaborators are aggressively engaged in over-expression and nano-particle drug delivery related studies of Wtmn to address the issues of enhanced-economic production, toxicity reduction and stability enhancement.

In conclusion we can say the production of Wtmn from *P. radicum* has been reported for the very first time in this report. The antifungal activity of Wtmn was re-confirmed using various fungal strains and it was found that Wtmn showed excellent antifungal activity in addition to its previously known anti-cancerous activity. *In silico* docking showed that the isolated Wtmn was capable of binding efficiently with various predicted fungal targets, like, 1FI4, 3A58 and 1S4N. Lower docking energies of Wtmn (1FI4: −7.69; 1S4N: −8.86; 3A58: −6.70) and presence of active amino acid residues (SER258 of 1FI4; SER 382, TYR380 of 1S4N and ASN106, CYS93 of 3A58) during its interaction with the predicted targets suggested that Wtmn works more efficiently than the commercially available antifungal drugs (voriconazole and nikkomycin) used in this computational study. Our *in-vitro* and *in- silico* findings suggest that Wtmn produced from *P. radicum* is a promising lead compound which could be potentially used as an efficient antifungal drug in humans after appropriate structural modifications to reduce toxicity and improve stability.

## Methods

### Isolation, characterization and fermentation conditions of microorganism

A fungal strain designated as SF, was isolated from the pre-treated soil sample collected from agricultural field of northern India [Geographical specifications: District- Faizabad, Uttar Pradesh, India (Latitude 26° 47’ N; Longitude 82° 12’ E)]. For the collection of soil sample no specific permissions were required for these locations/ activities and the performed field studies did not involve endangered or protected species. The strain was maintained on agar slants containing (g/L) dextrose 40, mycological peptone 10 and agar 20 (pH 7*–*7.2). The isolated strain was characterized morphologically and biochemically following the protocol given by Shirling and Gottlieb^36^, and Holt *et al.*^37^. Finally, the isolated strain was identified by 18S rRNA sequence homology.

Submerged fermentation was carried out in various production media [i.e., potato dextrose broth (PDB), sabouraud dextrose broth (SDB), czapekdox broth (CB), synthetic broth (SB) etc.] in Erlenmeyer flasks. Fermented broth from PDB showed maximum zone of inhibition against *Candida albicans* ATCC 24433 and considered as the most suitable medium for metabolite production (data not shown), and used as the production medium for further studies. Fermentation was carried out by inoculating the production medium with 1.0% (v/v) of 48 h old seed inoculums of the producer strain and incubating the flasks at 28 °C; 180 rpm for 120 h.

### Purification and chemical characterization of the active compound

Biomass was separated from the fermented culture by centrifuging it at 11,110xg for 20 min. The supernatant was extracted with chloroform and the organic layer containing active metabolites was collected and evaporated under reduced pressure. The residue was purified by silica gel (mesh size: 230–400) column chromatography using increasing gradient of methanol-chloroform (0–50%) as an elutant. The collected fractions were tested for antifungal activity by disc diffusion method against *Candida albicans* ATCC 24433. The fractions showing similar antifungal activity were pooled together and again subjected to silica gel column chromatography using mesh size 230–400. Purity of the fractions showing significant antifungal activity was further examined by high pressure liquid chromatography (HPLC) using analytical C-18 silica column (Lachrom) at a flow rate of 1 ml/min with Merck system at 254 nm. Finally the chemical structure of the compound was established with the help of UV (Perkin Elmer Lambda- 25 UV spectrophotometer), IR (FT-IR Perkin Elmer RX-1 spectrometer), ESMS (Micromass Quattro II triple quadrupole mass spectrometer) and ^1^H and ^13^C NMR spectra (600 MHz VARIAN INOVA instrument).

### Bioactivity of the purified compound

#### (a) Antifungal activity

Antifungal activity of the purified compound was quantitatively determined in terms of minimum inhibitory concentration (MIC) employing broth dilution method using 200 μg/ml as a starting concentration of the compound. MICs of the purified compound were studied with serial dilution method as recommended by the NCCLS guidelines^38^.

#### (b) Cytotoxic activity

Cytotoxic activity of the purified compound was evaluated using *in-vitro* MTT colorimetric assay against four human tumor cell lines (HEP-2 liver cells; IMR-32 neuroblastoma; HCT-15 and 502713 colon carcinoma cells) according to the methodology given by Mossman^39^.

### *In-silico* antifungal target site prediction

The *in-silico* studies were performed for the target site prediction for antifungal activity.

#### (a) Preparation of receptor

The crystal structures of the known potent antifungal drug targets were obtained from the Protein Data Bank (PDB)^40^. Analysis for number of missing atoms, removal of water molecules to clean the crystal structure and addition of H-atoms to these target proteins for correct ionization and tautomeric states of amino acid residues in three structures viz. 1FI4 (mevalonate-5-diphosphate decarboxylase), 1S4N (Glycolipid 2-alpha mannosyltransferase), 3A58 (exocyst complex component SEC3) were analysed for certain modifications which made them suitable for molecular docking. The energy was minimized for these protein structures using Gromacs software program (version 4.0) [Gromacs User Manual, Version 4.0 (2005); www.gromacs.org] in steepest descent followed by conjugate gradient method and all these minimization steps were performed using Gromos 87 force field^41^.

#### (b) Ligand preparation

The two-dimensional (2D) structures of ligand molecules or compounds (Wtmn, voriconazole, nikkomycin, pyridobenzimidazole) used in the present study have been shown in Table 4. The 2D structure of above mentioned compounds were retrieved from NCBI PubChem database and converted into 3D structures employing SYBYL X. The geometrics of these compounds were optimized using Merck Molecular Force Field (MMFF) and Gasteiger-Marsili Charge with 10,000 as maximum number of cycles, 0.01 as convergence criteria (root mean square gradient) and 1.0 as constant (medium’s dielectric constant which is 1 for in vacuo) in dielectric properties. The default values of electrostatic as 20.0, vdW as 10.0 and vdW after iterations as 10 Kcal/mol were used for non-bonded cut-off implemented in VLife MDS 1.0 (http://www.vlifesciences.com).

#### (c) Molecular docking set-up

The molecular docking studies were performed using open source software (AutoDock 4.2) and licensed version (FlexiDock) available in SYBYL. AutoDock combines energy evaluation through pre-calculated grids of affinity potential employing various search algorithms to find the suitable binding position for a ligand on a given protein^42^. Kollman united atom charges and polar hydrogens were added to the receptor PDB using AutoDock tools^42^. All rotatable bonds in the ligands were kept free to allow for flexible docking. Binding site for the ligand was chosen around Thr-262 and Ser-258 side chain which included all amino acid residues in the active site. Grid size was set to 60 × 60 × 60 grid points (x, y and z), with spacing between the grid points kept at 0.375 Å. The Lamarckian genetic algorithm was chosen for searching the best conformers. The standard docking protocol was applied which is based on the population size of 150 randomly placed individuals, a maximum number of 250000 energy evaluations, a mutation rate of 0.02, a crossover rate of 0.80 and an elitism value of 1. Twenty five independent docking runs were carried out for each inhibitor and cluster tolerance was kept at 1.0 Å. The best docking conformers from each class of ligand were further verified using FlexiDock available from SYBYL-X, each FlexiDock simulation was performed with 25,000 generations. Binding site was defined around 3 Å region of previously docked ligand, the resulting conformations from FlexiDock runs were compared with AutoDock to establish veracity of AutoDock runs.

#### (d) Molecular dynamics set-up

Based upon the molecular docking outcomes, molecular dynamics simulations of active site enzyme- inhibitor complexes were carried out using Gromacs 4.2 suite of programs (www.gromacs.org) using AMBER 94 force field (www.ambermd.org). Each of the complexes comprising the active site was placed in the centre of 60*60*60 Ǻ cubic box and solvated by SPC/E water molecules^41^. The gromacs topology file for ligands was generated using the PRODRG2 server. The time constant for Berendsen temperature coupling and Berendsen pressure coupling were both set at 0.1. The molecular dynamics method was done at a constant temperature and pressure of 300 K and 1 bar. All of the complexes were energy minimized using steepest descent method. Further, a 30 ps position restraining simulation was carried out to restrict the movement of the protein in the simulation. For the long range electrostatic interactions, Particle Mesh Ewald (PME) electrostatic was used. The cut-off for coulomb interactions and Van der Waal interactions were set to 1.0 nm and 1.4 nm, respectively. The LINCS algorithm was employed for all the bond constraints.

## Additional Information

**How to cite this article**: Singh, V. *et al.* Isolation, characterization and antifungal docking studies of wortmannin isolated from Penicillium radicum. *Sci. Rep.* **5**, 11948; doi: 10.1038/srep11948 (2015).

## Supplementary Material

Supplementary Information

## Figures and Tables

**Table 1:** Biochemical properties of the isolated strain.

**S. No.**	**Physiological properties**	**Response**
1	Growth on different medium	
	Glycerol Nitrate Agar (GNA)	Thin light brown, conidia absent
	Malt Extract Agar (MEA)	light yellow with yellowish green conidia
	Potato Dextrose Agar (PDA)	Olive green with olive green conidia
	Czapek’s Dox + Yeast Extract Agar (CYA)	Floccose, bright yellow with greenish yellow conidia
	Sabouraud Dextrose Agar (SDA)	Floccose, white with yellowish conidia
	Corn Meal Agar (CMA)	Floccose, pale white with dark yellow conidia
	Oat Meal Agar (OMA)	Thin mat like light brown, conidia absent
	Glucose-yeast extract-malt extract (GYM) Agar	Velutinous, cream coloured with dirty yellow conidia
2	Enzyme production	
	Urease	Positive
	Protease	Positive
	Tyrosinase	Positive
	Amylase	Positive
	Chitinase	Negative
	Esterase	Positive
	Lipase	Negative
3.	Citrate utilization	Negative
4.	Milk coagulation	Positive
5.	Sensitivity for antifungal drugs	
	Kt^100^	R
	Ap^100^	R
	Ns^100^	R
	It^100^	R
	Fu^100^	R
	Ec^100^	S
	Mc^100^	S

Note: Kt: Ketoconazole; Ap: Amphotericin B; Ns: Nystatin; It: Itraconozole; Fu: Fluconzole; Ec: Econazole-nitrate; Mc: Miconazole.

**Table 2:** Antifungal activity of Wtmn isolated from *Penicillium radicum* MTCC 7584.

**S. No.**	**Test strain**	**Antifungal drugs MIC** **±** **SD (μg/ml)**		
		*Wtmn*	*Amphotericin B*	*Fluconazole*
1	*Candida albicans* ATCC 24433	0.39 ± 0.05	0.39 ± 0.02	6.25 ± 0.44
2	*Candida albicans* ATCC 10231	3.13 ± 0.44	1.56 ± 0.11	10.2 ± 0.72
3	*Candida albicans* ATCC18804	1.56 ± 0.22	1.56 ± 0.11	12.5 ± 0.88
4	*Candida albicans* ATCC 2091	12.5 ± 1.76	1.56 ± 0.11	25 ± 1.76
5	*Candida albicans* ATCC 90028	1.56 ± 0.22	1.56 ± 0.11	12.5 ± 0.88
6	*Candida tropicalis* ATCC 750	12.5 ± 1.76	6.25 ± 0.44	25 ± 1.76
7	*Cryptococcus terreus* ATCC 11799	1.56 ± 0.22	12.5 ± 0.88	12.5 ± 0.88
8	*Trichophyton rubrum* ATCC 296	1.56 ± 0.22	3.13 ± 0.22	6.25 ± 0.44
9	*Alternaria alternata* ATCC 6663	3.13 ± 0.44	3.13 ± 0.22	6.25 ± 0.44
10	*Rhizoctonia oryzae* ATCC 52545	0.78 ± 0.11	2.5 ± 0.17	5.0 ± 0.35
11	*Aspergillus terreus* DSM 826	10 ± 1.41	1.25 ± 0.08	12.5 ± 0.88
12	*Aspergillus versicolor* DSM 1943	0.5 ± 0.07	1.25 ± 0.08	10 ± 0.70
13	*Aspergillus niger* DSM 2182	12.5 ± 1.76	2.5 ± 0.17	12.5 ± 0.88
14	*Aspergillus niger* DSM 63263	25 ± 3.53	5.0 ± 0.35	12.5 ± 0.88
15	*Aspergillus fumigates* ATCC 204305	3.13 ± 0.44	3.13 ± 0.22	6.25 ± 0.44
16	*Aureobasidium pullulans* DSM 2404	10 ± 1.41	5.0 ± 0.35	12.5 ± 0.88
17	*Fusarium moniliforme* ATCC 14164	5.0 ± 0.70	5.0 ± 0.35	25 ± 1.76
18	*Saccharomyces cerevisiae* ATCC 2365	1.56 ± 0.22	1.56 ± 0.11	6.25 ± 0.44

**Table 3:** Cytotoxic activity of various compounds against various cancerous cell lines.

**Compounds**	**Concentration (μg/ml)**	**Cell lines**			
		**Liver**	**Neuroblastoma**	**Colon**	
		**HEP-2**	**IMR-32**	**HCT-15**	**502713**
		**Growth inhibition (%) ± SD**			
SF (Wtmn)	10	49 ± 0.71	−	74 ± 1.41	76 ± 1.41
SF (Wtmn)	30	52 ± 1.41	−	91 ± 2.12	89 ± 2.82
Actinomycin D	10	66 ± 2.83	68 ± 0.71	70 ± 0.71	69 ± 0.71
Actinomycin D	30	82 ± 1.41	87 ± 0.71	72 ± 1.76	98 ± 2.12
Mito-C	3.34	82 ± 0.71	87 ± 0.71	−	86 ± 2.47
Adriamycin	0.54	79 ± 0.71	89 ± 1.41	−	22 ± 2.12
5-Fu	6.12	−	−	27 ± 1.41	15 ± 2.12

Note: (−): No activity

**Table 4:**
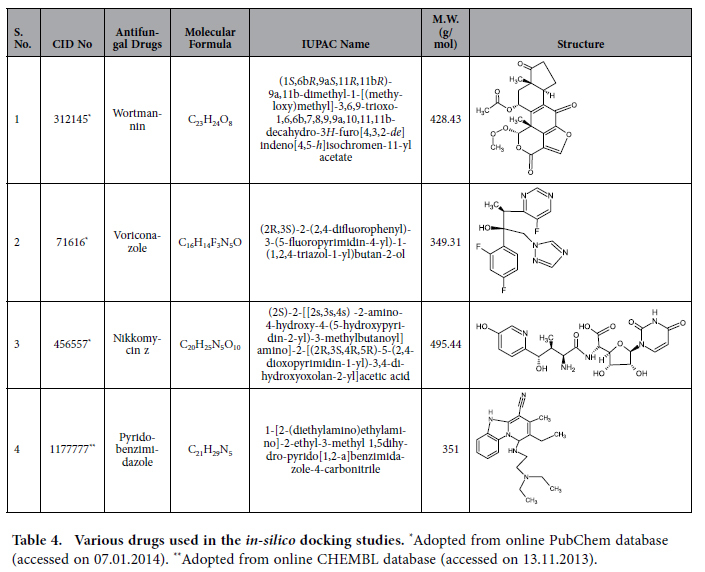
Various drugs used in the *in-silico* docking studies.

**Table 5:** The interaction energies (kcal*/*mol) of antifungal compounds with protein using AutoDock.

**PDB ID**	**Antifungal compounds**	**Binding energy(kcal/mol)**	**Docked energy(kcal/mol)**	**Internal energy(kcal/mol)**	**Intermolecular energy(kcal/mol)**	**Torsional energy(kcal/mol)**	**RMSD(Å)**
**1FI4**	Wortmannin	−6.01	−7.69	−0.44	−7.25	1.25	65.866
	Voriconazole	−4.88	−5.73	0.70	−6.43	1.56	60.273
	Nikkomycin	−3.43	−6.27	−0.04	−6.23	2.80	63.640
	pyridobenzimidazole	−7.88	−10.24	−0.18	−10.06	2.18	67.175
**1S4N**	Wortmannin	−7.28	−8.86	−0.34	−8.53	1.25	72.258
	Voriconazole	−5.22	−5.67	1.11	−6.78	1.56	75.792
	Nikkomycin	−4.83	−7.79	−0.15	−7.63	2.80	73.499
	pyridobenzimidazole	−19.81	−22.36	−0.37	−21.99	2.18	76.745
**3A58**	Wortmannin	−5.48	−6.70	0.02	−6.73	1.25	67.693
	Voriconazole	−6.29	−6.34	1.56	−7.85	1.56	89.860
	Nikkomycin	−3.06	−5.89	−0.03	−5.86	2.81	83.461
	pyridobenzimidazole	−5.83	−8.41	−0.40	−8.01	2.18	85.983

**Table 6:** Predicted amino acid residues in protein templates using molecular docking methods.

**PDB ID**	**Compound**	**Amino acid residues in H-bond**	**Amino acids residues in antifungal proteins**
**1FI4**	Wortmannin	SER-258	THR262, SER258, MSE255, VAL229, LEU221
	Voriconazole	ALA303	ASP205, ALA303, GLY304, ASP201, GLY 112, SER 207
**1S4N**	Wortmannin	SER382,TYR380	SER382, TYR380, HIS379, ILE378, LEU431, GLN429
	Pyridobenzimidazole	SER382	ASP383, HIS379, GLU396, TYR426, LEU431, SER382
**3A58**	Wortmannin	ASN106,CYS93	GLU103, ASP98, CYS93, LYS100, ASN106
	Nikkomycin	ARG96,CYS93	ARG96, PRO105, GLU103, ASP98, ILE166, CYS92, ASP98

**Table 7:** Comparative docking energies (AutoDock & FlexiDock) of antifungal drugs and their interaction with the active site.

**PDB ID**	**Inhibitors**	**Docking energy AutoDock (Kcal/mol)**	**Docking energy FlexiDock (Kcal/mol)**	**Active site residues**
1FI4	Wortmannin	−7.69	−697.214	Ser 258
	Voriconazole	−5.73	−505.913	Ala 303
	Nikkomycin	−6.27	−498.682	Arg 96
	Pyriobenzimidazole	−10.24	−712.429	Ser 382
1S4N	Wortmannin	−8.99	−358.968	Ser 358
	Voriconazole	−9.20	−412.359	Ala 303, Asp205
	Nikkomycin	−8.92	−406.322	Arg 96, Cys 93
	Pyriobenzimidazole	−22.36	−908.254	Ser 382, Asp 383
3A58	Wortmannin	−6.70	−294.647	Asp 214, Val 78
	Voriconazole	−6.34	−532.144	Asp 34,Val 78
	Nikkomycin	−5.89	−438.214	Asp 214,Ser 218
	Pyriobenzimidazole	−8.41	−798.385	Asp 383, Asp 383

## References

[b1] Meyer V. A small protein that fights fungi: AFP as a new promising antifungal agent of biotechnological value. Appl. Microbiol. Biotechnol. 78, 17–28 (2008). 18066545 10.1007/s00253-007-1291-3

[b2] Montague J. A. & Bender G. L. (inventors), Nutrient status of plants in soils. U.S. Patent 5,770,787. (http://patent.ipexl.com/US/05770787.html (1998) Date of access: 02/04/2015).

[b3] Wakelin S. A., Ryder M. H. & Warren R. A. Effect of soil properties on growth promotion of wheat by *Penicillium radicum*. Aust. J. Soil Res. 42, 897–904 (2004).

[b4] Vining L. C. Functions of secondary metabolites. Annu. Rev. Microbiol. 44, 395–427 (1990). 2252388 10.1146/annurev.mi.44.100190.002143

[b5] Ingroff A. E. Novel antifungal agents, targets or therapeutic strategies for the treatment of invasive fungal diseases: a review of the literature (2005-2009). Rev. Iberoam. Micol. 26, 15–22 (2009). 19463273 10.1016/S1130-1406(09)70004-X

[b6] Arcaro A. & Wymann M. P. Wtmn is a potent phosphatidylinositol 3-kinase inhibitor: the role of phosphatidylinositol 3,4,5-trisphosphate in neutrophil responses. Biochem. J. 296, 297–301 (1993). 8257416 10.1042/bj2960297 PMC1137693

[b7] Fukao T. & Koyasu S. PI3K and negative regulation of TLR signaling. Trends Immunol. 24, 358–363 (2003). 12860525 10.1016/s1471-4906(03)00139-x

[b8] Hazeki K. *et al.* Opposite effects of Wtmn and 2-(4-Morpholinyl)-8-phenyl-1(4H)-benzopyran-4-one hydrochloride on toll- like receptor-mediated nitric oxide production: negative regulation of nuclear factor- {kappa}B by phosphoinositide 3-kinase. Mol. Pharmacol. 69, 1717–1724 (2006). 16474002 10.1124/mol.105.021162

[b9] Blommaart E. F., Krause U., Schellens J. P., Vreeling-Sindelárová H. & Meijer A. J. The phosphatidylinositol 3-kinase inhibitors Wtmn and LY294002 inhibit autophagy in isolated rat hepatocytes. Eur. J. Biochem. 243, 240–246 (1997). 9030745 10.1111/j.1432-1033.1997.0240a.x

[b10] Weng L. P., Brown J. L. & Eng C. PTEN induces apoptosis and cell cycle arrest through phosphoinositol-3-kinase/Akt-dependent and independent pathways. Hum. Mol. Genet. 10, 237–242 (2001). 11159942 10.1093/hmg/10.3.237

[b11] Karve S. *et al.* Revival of the abandoned therapeutic wortmannin by nanoparticle drug delivery. Proc. Natl. Acad. Sci. USA 109, 8230–8235 (2011). 22547809 10.1073/pnas.1120508109 PMC3361429

[b12] Wipf P. & Halter R. J. Chemistry and biology of wortmannin. Org. Biomol. Chem. 3, 2053–2061 (2005). 15917886 10.1039/b504418a

[b13] Huang S. Y. & Zou X. Efficient molecular docking of NMR structures: Application to HIV-1 protease. Protein Sci. 16, 43–51 (2007). 17123961 10.1110/ps.062501507 PMC2222846

[b14] Fukunishi Y. & Nakamura H. Prediction of ligand-binding sites of proteins by molecular docking calculation for a random ligand library. Protein Sci. 20, 95–106 (2011). 21064162 10.1002/pro.540 PMC3047065

[b15] McPhillie M. J. *et al.* Computational methods to identify new antibacterial targets. Chem Biol Drug Des 85, 22–29 (2015). 24974974 10.1111/cbdd.12385

[b16] Singh V., Tripathi C. K. M.& Bihari V. Production, optimization and purification of an antifungal compound from *Streptomyces capoamus* MTCC 8123. Med. Chem. Res. 17, 94–102 (2008).

[b17] Silverstein R. M. & Webster F. X. Spectrometric identification of organic compounds. 6^th^ edn, (Wiley, Canada, 1998).

[b18] MacMillan J., Vanstone A. E. & Yeboah S. K. The structure of wortmannin, a steroidal fungal metabolite. Chem. Commun. 613–614 (1968). 10.1039/C19680000613.

[b19] Brian P. W., Curtis P. J., Hemming H. G. & Norris G. L. F. Wortmannin, an antibiotic produced by *Penicillium wortmanni*. T. Brit. Mycol. Soc. 40, 365–368 (1957).

[b20] Petcher T. J., Weber H. P. & Kis Z. Crystal structure and absolute configuration of wortmannin and of wortmannin p-bromobenzoate. J. Chem. Soc., Chem. Commun. 19, 1061–1062 (1972).

[b21] MacMillan J., Vanstone A. E. & Yeboah S. K. Fungal products. Part III. Structure of wortmannin and some hydrolysis products. J. Chem. Soc., Perkin Trans. 1, 2898–2903 (1972).

[b22] Abbas H. K. & Mirocha C. J. Isolation and purification of a hemorrhagic factor (Wortmannin) from *Fusarium oxysporum* (N17B). Appl. Environ. Microbiol. 54, 1268–1274 (1988). 3389818 10.1128/aem.54.5.1268-1274.1988 PMC202638

[b23] Nakanishi S. *et al.* Wortmannin, a microbial product inhibitor of myosin light chain kinase. J. Boil. Chem. 267, 2157–2163 (1992). 1733924

[b24] Samson R. A. *et al.* Phylogeny and nomenclature of the genus Talaromyces and taxa accommodated in *Penicillium* subgenus *Biverticillium*. Stud. Mycol. 70, 159–83 (2011). 22308048 10.3114/sim.2011.70.04 PMC3233910

[b25] Hocking A. D., Whitelaw M. & Harden T. J. Penicillium radicum sp. nov. from the rhizosphere of Australian wheat. Mycol. Res. 102, 801–806 (1998).

[b26] Schultz R. M. *et al.* *In vitro* and *in vivo* antitumor activity of the phosphatidylinositol-3-kinase inhibitor, wortmannin. Anticancer Res. 15, 1135–1139 (1995). 7653991

[b27] Bonanno J. B. *et al.* Structural genomics of enzymes involved in sterol/isoprenoid biosynthesis. Proc. Natl. Acad. Sci. USA 98, 12896–12901 (2001). 11698677 10.1073/pnas.181466998 PMC60796

[b28] Lobsanov Y. D. *et al.* Structure of Kre2p/Mnt1p: a yeast alpha1,2-mannosyltransferase involved in mannoprotein biosynthesis. J. Biol. Chem. 279, 17921–17931 (2004). 14752117 10.1074/jbc.M312720200

[b29] Yamashita M. *et al.* Structural basis for the Rho- and phosphoinositide-dependent localization of the exocyst subunit Sec3. Nat. Struct. Mol. Biol. 17, 180–186 (2010). 20062059 10.1038/nsmb.1722

[b30] Singh V. & Somvanshi P. Homology modeling of 3-oxoacyl-acyl carrier protein synthase II from *Mycobacterium tuberculosis* H37Rv and molecular docking for exploration of drugs. J. Mol. Model. 15, 453–460 (2009). 19083031 10.1007/s00894-008-0426-5

[b31] Singh V., Somvanshi P., Rathore G., Mishra B. N. & Kapoor D. Gene cloning and expression of hemolysin gene from Aeromonas hydrophila. Prot. Express Puri. 65, 1–7 (2009). 10.1016/j.pep.2008.11.015 19136063

[b32] Singh V., Somvanshi P., Rathore G., Kapoor D. & Mishra B. N. Gene cloning, expression and characterization of recombinant aerolysin from *Aeromonas hydrophila*. Appl. Biochem. Biotechnol. 160, 1985–1991 (2010). 19763901 10.1007/s12010-009-8752-3

[b33] Singh V. & Somvanshi P. Toward the virtual screening of potential drugs in the homology modeled NAD^+^ dependent DNA ligase from *Mycobacterium tuberculosis*. Prot. Peptide Lett. 17, 269–276 (2010). 10.2174/092986610790225950 20214650

[b34] Singh V. & Somvanshi P. Inhibition of oligomerization of aerolysin from *Aeromonas hydrophila*: Homology Modeling and docking approach for exploration of hemorrhagic septicemia. Lett. Drug Des. Disc. 6, 215–223 (2009).

[b35] Sinha S., Tandon A. & Srivastava D. S. Inhibitory activity of Imidazole with cytochrome P 450 2B4: A Docking and Molecular Dynamics Study. Biofrontiers 1, 24–31 (2010).

[b36] Shirling E. B. & Gottlieb D. Methods for characterization of *Streptomyces* sp. Int. J. Syst. Bacteriol. 16, 313–340 (1966).

[b37] Holt J. G., Krieg N. R., Sneath P. H. A., Staley J. T. & Williams S. T. Bergey´s manual of determinative bacteriology, 9th edn, (Williams & Wilkins, Baltimore, 1996).

[b38] National Committee for Clinical Laboratory Standards. Approved standard M7-A5 Methods for dilution antimicrobial susceptibility tests for bacteria that grow aerobically. 4^th^ edn. (Wayne, PA: NCCLS, 2000).

[b39] Mossman T. Rapid calorimetric assay for cellular growth and survival: application to proliferation and cytotoxic assays. J. Immunol. Met. 65, 55–63 (1983). 10.1016/0022-1759(83)90303-4 6606682

[b40] Asojo O. A. *et al.* Structures of Ser205 mutant plasmepsin II from *Plasmodium falciparum* at 1.8 A in complex with the inhibitors rs367 and rs370. Acta Crystallogr. D. Biol. Crystallogr. 58, 2001–2008 (2002). 12454457 10.1107/s0907444902014695

[b41] Hess B., Kutzner C., Spoel D. V. & Lindahl E. GROMACS 4: Algorithms for highly efficient, load-balanced, and scalable molecular simulation. J. Chem. Theory Compt. 4, 435–447 (2008). 10.1021/ct700301q 26620784

[b42] Morris G. M. *et al.* AutoDock4 and AutoDockTools4: Automated docking with selective receptor flexibility. J. Comput. Chem. 30, 2785–2791 (2009). 10.1002/jcc.21256. 19399780 PMC2760638

