# Supplementary material for: Isolation, characterization and antifungal docking studies of wortmannin isolated from Penicillium radicum
Source: Sci Rep. 2015 Jul 10;5:11948. doi: 10.1038/srep11948 (PMC4498184; doi:10.1038/srep11948)
Supplement: Supplementary Information [file srep11948-s1.pdf]

Isolation, characterization and antifungal docking studies of wortmannin isolated from *Penicillium radicum*

Vineeta Singh[1]*, Vandana Praveen[2], Divya Tripathi[3], Shafiul Haque[4,5], Pallavi Somvanshi[6], S.B. Katti[7], C.K.M. Tripathi[2]

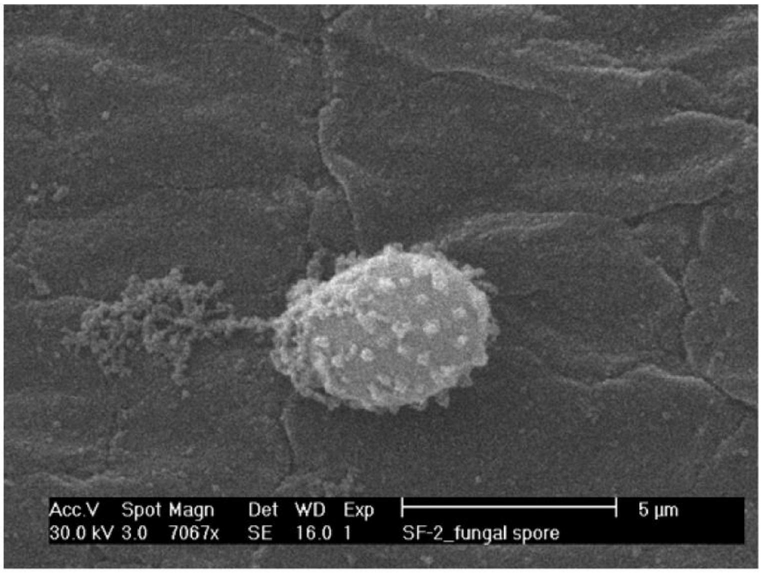

Figure SI1: Scanning Electron Microscopy of the bacterial spore

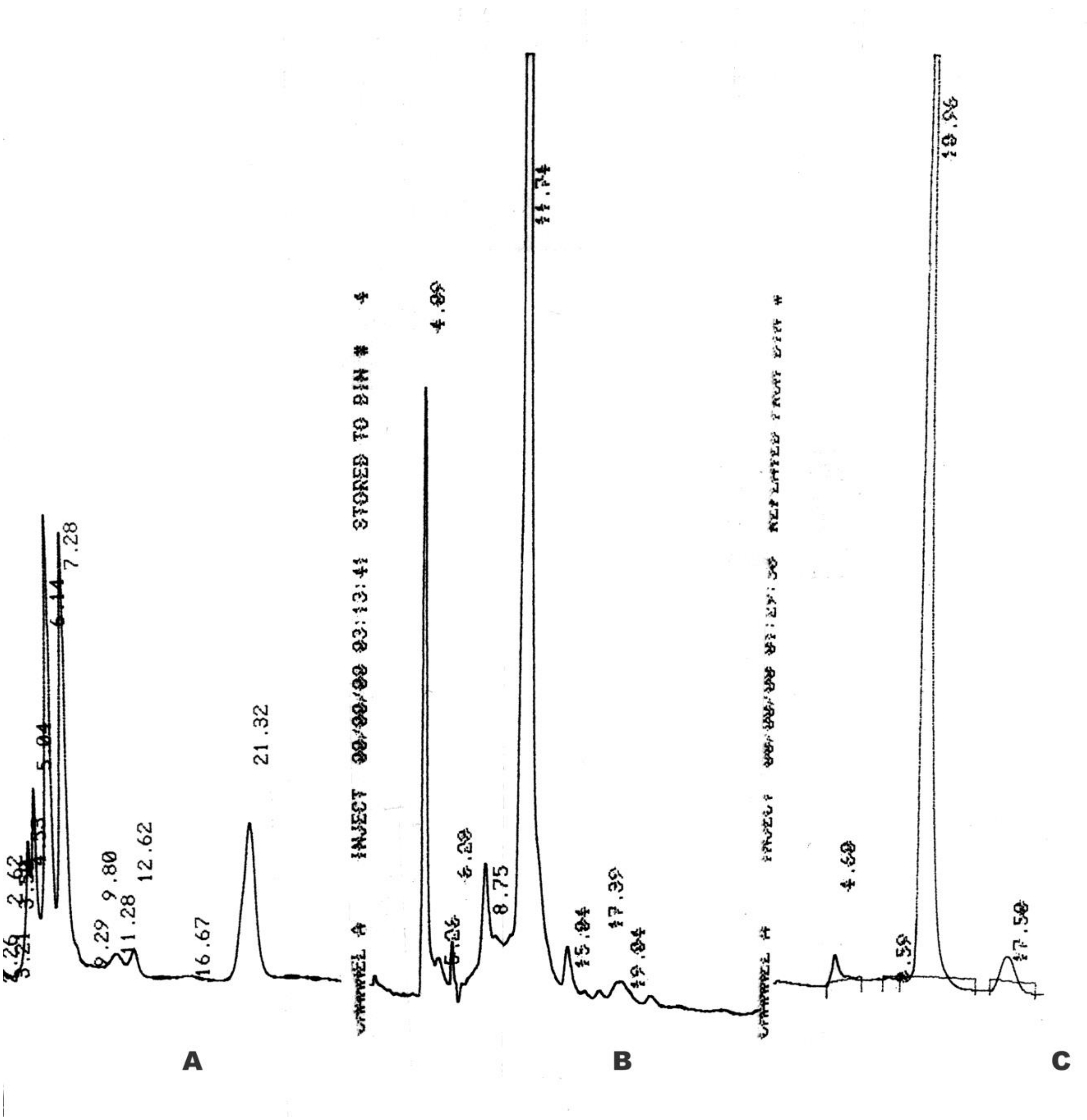

Figure SI2: HPLC profiles of SF broth showing: [A] crude extract; [B] active fraction eluted from Silica-gel column and [C] B washed with methanol; Peak at RT 10.96 showed activity against fungi as well as cancer cell lines.

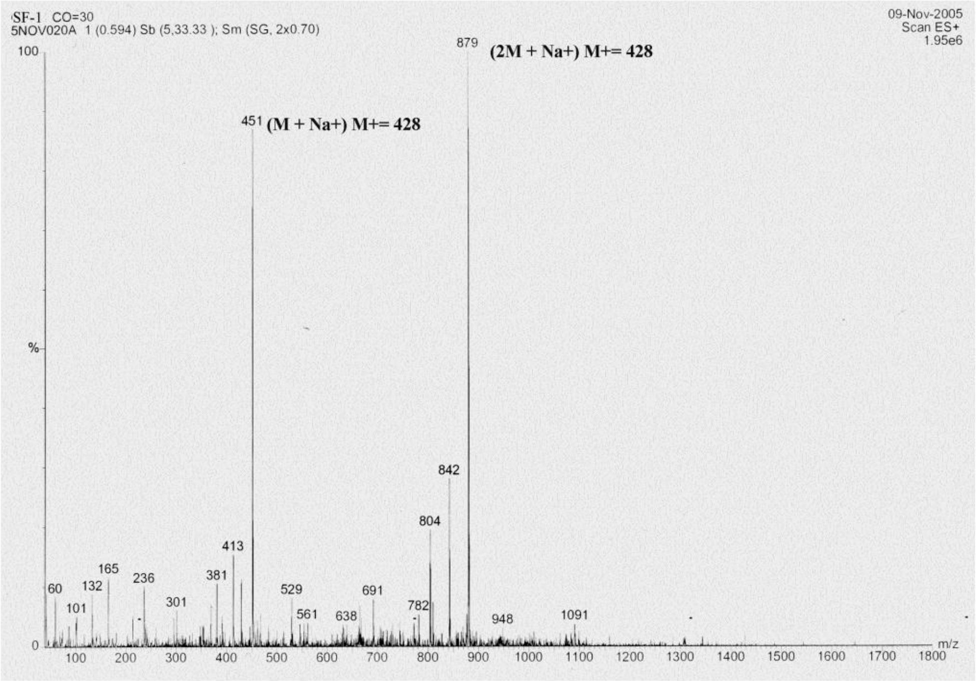

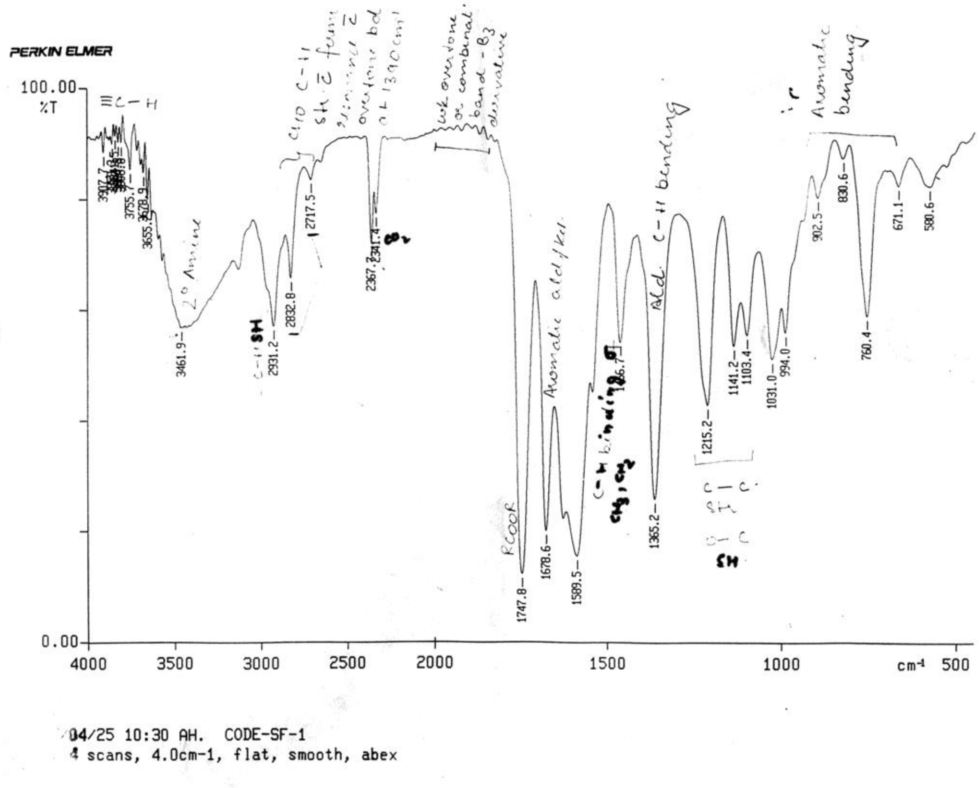

Figure SI3: Mass (ESI-MS) and Infrared spectra (IR) of antifungal compound from *P. radicum*

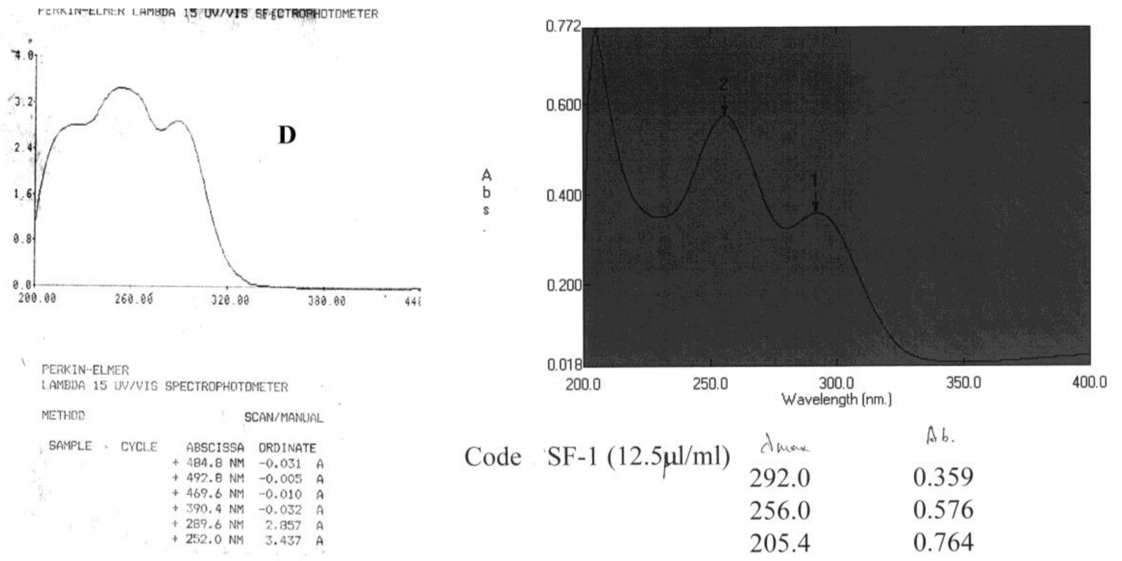

Figure SI4: UV/VIS spectra- Crude from SF and E. pure and active compound from SF (RT 11.98 min.), SF-1

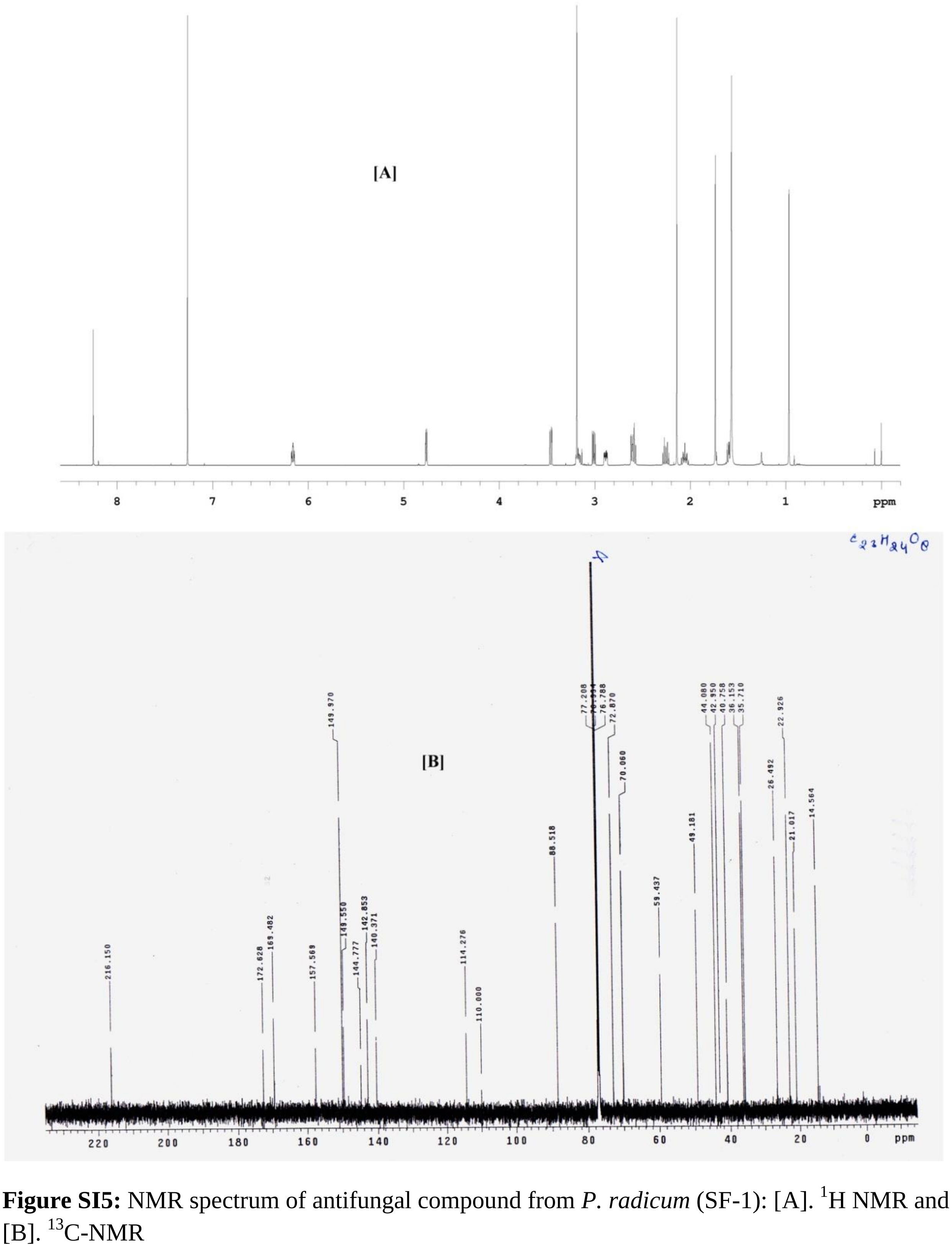

Figure SI5: NMR spectrum of antifungal compound from *P. radicum* (SF-1): [A]. ^{1}H NMR and [B]. ^{13}C-NMR

Table SI1. ^{1}H and ^{13}C NMR findings

Carbon number	Group	Shifts (δ) ppm ^{13}C	Shifts (δ) ppm ^{1}H
C_1	CH	88.518	4.76, dd (1.83, 6.96)
C_2	CH_2	72.870	H_A: 3.46, dd (1.95. 11.23);<br>H_B: 3.01, dd (7.01, 11.09)
C_3	CO	127.569	
C_4	C	114.276	
C_5	C	142.853	
C_6	C	144.777	
C_7	CO	172.628	
C_8	C	140.371	
C_9	C	149.550	
C_{10}	C	40.758	
C_{11}	CH	70.060	6.16, ddd (2.77, 7.53, 8.93)
C_{12}	CH_2	36.153	H^A: 2.59, m;<br>H^B: 1.60, dd (8.7, 12.8) + residual water
C_{13}	C	49.181	
C_{14}	CH	44.080	2.89, ddd (2.66, 5.97, 12.68)
C_{15}	CH_2	22.926	H^A: 3.17, m;<br>H^B: 2.06, m
C_{16}	CH_2	35.710	H^A: 2.59, m;<br>H^B: 2.26, m
C_{17}	CO	216.150	
C_{18}	CH_3	14.564	0.97, s
C_{19}	CH_3	26.492	1.74, s
C_{20}	CH	149.970	8.25, s
C_{21}	CO	169.482	
C_{22}	CH	21.017	2.14, s
C_{23}	CH	59.437	3.19, s
